# Breed Differences in Pig Liver Esterase (PLE) between Tongcheng (Chinese Local Breed) and Large White Pigs

**DOI:** 10.1038/s41598-018-34695-y

**Published:** 2018-11-05

**Authors:** Qiling Xiao, Qiongqiong Zhou, Lu Yang, Zhongyuan Tian, Xiliang Wang, Yuncai Xiao, Deshi Shi

**Affiliations:** 1 State Key Laboratory of Agricultural Microbiology, College of Veterinary Medicine, Huazhong Agricultural University, Wuhan, 430070 Hubei China; 2 Key Laboratory of Development of Veterinary Diagnostic Products of Ministry of Agricultural, College of Veterinary Medicine, Huazhong Agricultural University, Wuhan, 430070 Hubei China; 3 The Cooperative Innovation Center for Sustainable Pig Production, Wuhan, 430070 Hubei China

## Abstract

Human carboxylesterases has been proven to be age and race-related and a sound basis of clinical medication. PLE involve in signal transduction and highly catalyze hydrolysis. Therefore, the expression level of PLE most probably exist age and breed difference and lead to significant differences of pharmacology and physiology. Four age groups of Tongcheng (TC) and Large White (LW) pigs were selected to explore PLE breed and age differences, and it was found that PLE mRNA was most abundant in liver in both breeds. In liver, PLE levels and hydrolytic activities increased with age, and PLE levels (except for 3 month) and the hydrolytic activities were higher in LW than in TC across all age groups. Abundance of PLE isoenzymes was obvious different between breeds and among age groups. The most abundant PLE isoenzyme in LW and TC pigs was PLE-A1 (all age groups) and PLE-B9 (three early age groups) or PLE-G3 (adult groups), respectively. 103 new PLE isoenzymes were found, and 55 high-frequency PLE isoenzymes were accordingly classified into seven categories (A-G). The results of this research provide a necessary basis not only for clinical medication of pigs but also for pig breeding purposes.

## Introduction

Carboxylesterases(E.C.3.1.1.1) belong to the serine hydrolase family, they catalyze the hydrolysis of endogenous and exogenous compounds containing carboxylic acid esters, amides, and thioesters^1,2^. These enzymes play critical roles in drug metabolism^2–8^, lipid mobilization^1,9–12^, and pesticide detoxification^13–16^. Studies on human and rodents have found that carboxylesterases control the metabolism and detoxification of nearly one-third of the drugs ingested. Expression level of human and mouse carboxylesterases has been proven to be age-related, the expression profiles and activities of carboxylesterases have a very significant impact on metabolism, efficacy, and safety of drugs; additionally, their expression spectrum and racial differences have become the basis for clinical medication^5,7,17–24^.

The expression of pig carboxylesterases is observed in various organs, being the most abundant in the liver, hence, known as PLE. Only the trimer structure of PLE has enzyme active, and in the very early studies, the subunits of the trimer structure were roughly classed as α-, β- and γ-subunits according to the difference in molecular weight and isoelectric point^25–27^. According to isoelectric point, enzyme kinetics, and substrate specificity, Junge and Heymann(1979) isolated four kinds of PLE trimmers, named γγγ, αγγ, ααγ, and ααα^26^. PLE extracted from pig liver is a mixture of multiple isozymes of PLE, and it is difficult to obtain a single and highly purified enzyme using physical or chemical methods, because of the high degree of similarity of physical and chemical properties among PLE isozymes. Fortunately, not only a single PLE of high purity has been obtained by genetic engineering methods but also the cDNA sequence and the polypeptide sequence of different subunits of PLE and differences in the enzymatic activity have been documented^28–31^. Structure and genomics of PLE gene families have also been studied by our research group. The PLE gene spans 30 kb containing 14 exons, most of which are highly conserved, and 13 introns^32^. The full length of PLE encoding cDNA has 1698bp, and it encodes a polypeptide of 566 amino acid (AA) residues. The N-terminal is an 18 AA peptide signal-sequence and C-terminal tetrapeptides HAEL (His-Ala-Glu-Leu) are considered as an endoplasmic reticulum retention signal^28,31,33^. The C-terminal tetra peptides HXEL bind to the KDEL receptor, thus locating the carboxylate ester in the endoplasmic reticulum^34^. PLE possesses high hydrolytic activity of rich sources, broad substrate specificity, and especially high enantioselectivity and stereoselectivity of hydrolysis, which makes PLE one of the most important hydrolases in the field of organic synthesis^35–39^, whereby for one century the study of PLE has focused on its application to organic synthesis. A few research results have suggested that PLE is involved in body signal transduction^28,40^ and hydrolysis of endogenous and exogenous compounds^1,28,40–44^, while its pharmacological, toxicological, and physiological functions have received very little attention.

Based on the above mentioned, it is reasonable to speculate that PLE plays an important role in the pharmacological and physiological effects of drug treatments; moreover, PLE expression differences at different ages and in different breeds may lead to pharmacology, toxicology, and physiologic differences.

In order to obtain the necessary data for clinically rational drug use and to explore PLE pharmacology and physiology roles, in this study, different age groups of LW pigs and TC pigs were selected to study the expression profile and breed differences in PLE. Differences in total level, abundance, and enzyme activity among PLE isoenzymes from two breeds and four different age groups were systematically studied.

## Results

### PLE isoenzymes mRNA was most abundant in the liver and followed by the kidney, small intestine, and skin

**Figure 1:**
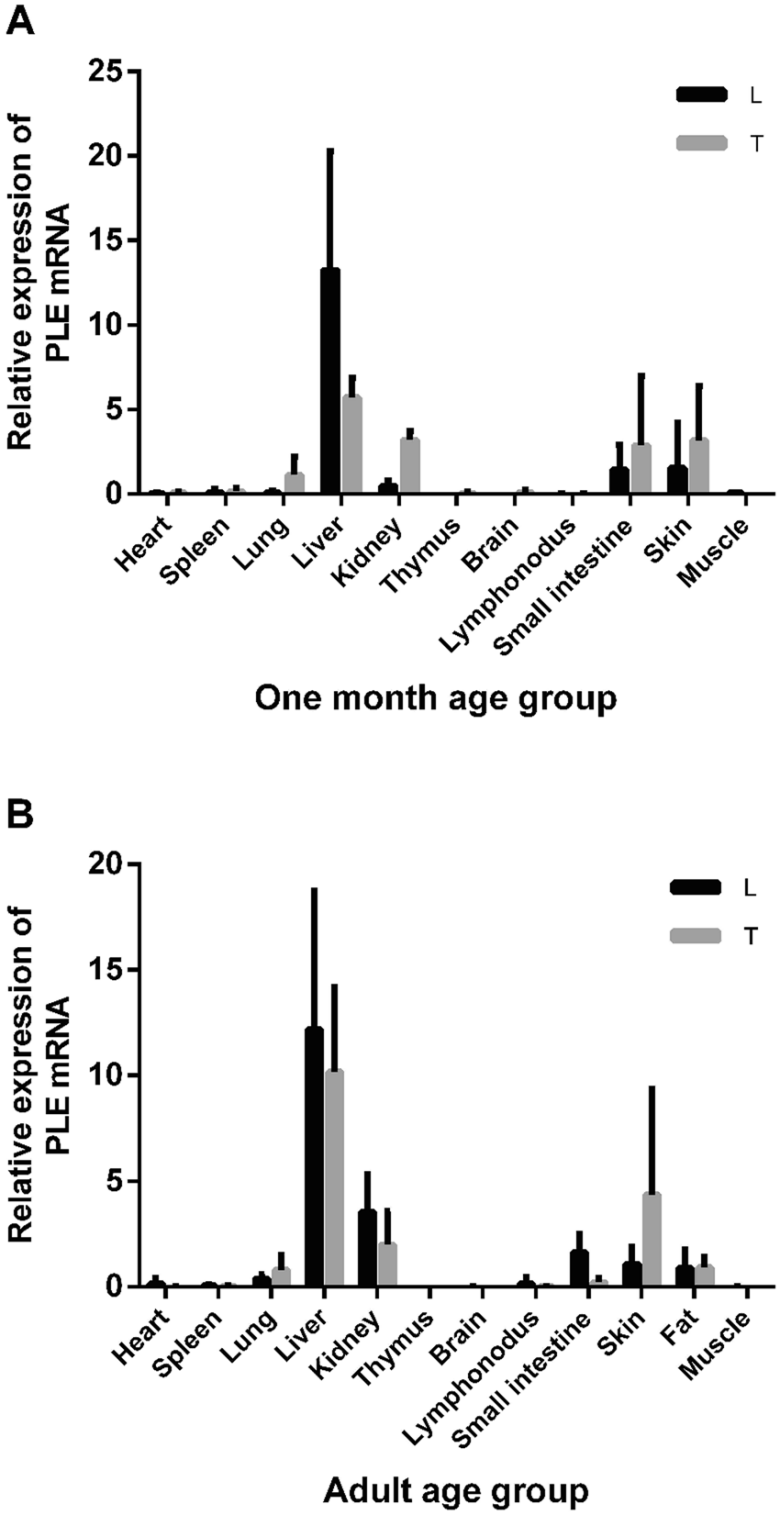
PLE mRNA level in different tissues from one month and adult age groups of LW pigs (L) and TC pigs (T). PLE isoenzymes mRNA was most abundant in the liver and followed by the kidney, small intestine, and skin from one month (**A**) and adult age groups (**B**) of two breeds. Total RNA was subjected to quantitative real-time PCR (qRT-PCR) analysis of PLE mRNA by SYBR. The PLE mRNA levels were normalized by GAPDH. The data are means (±SD).

**Figure 2:**
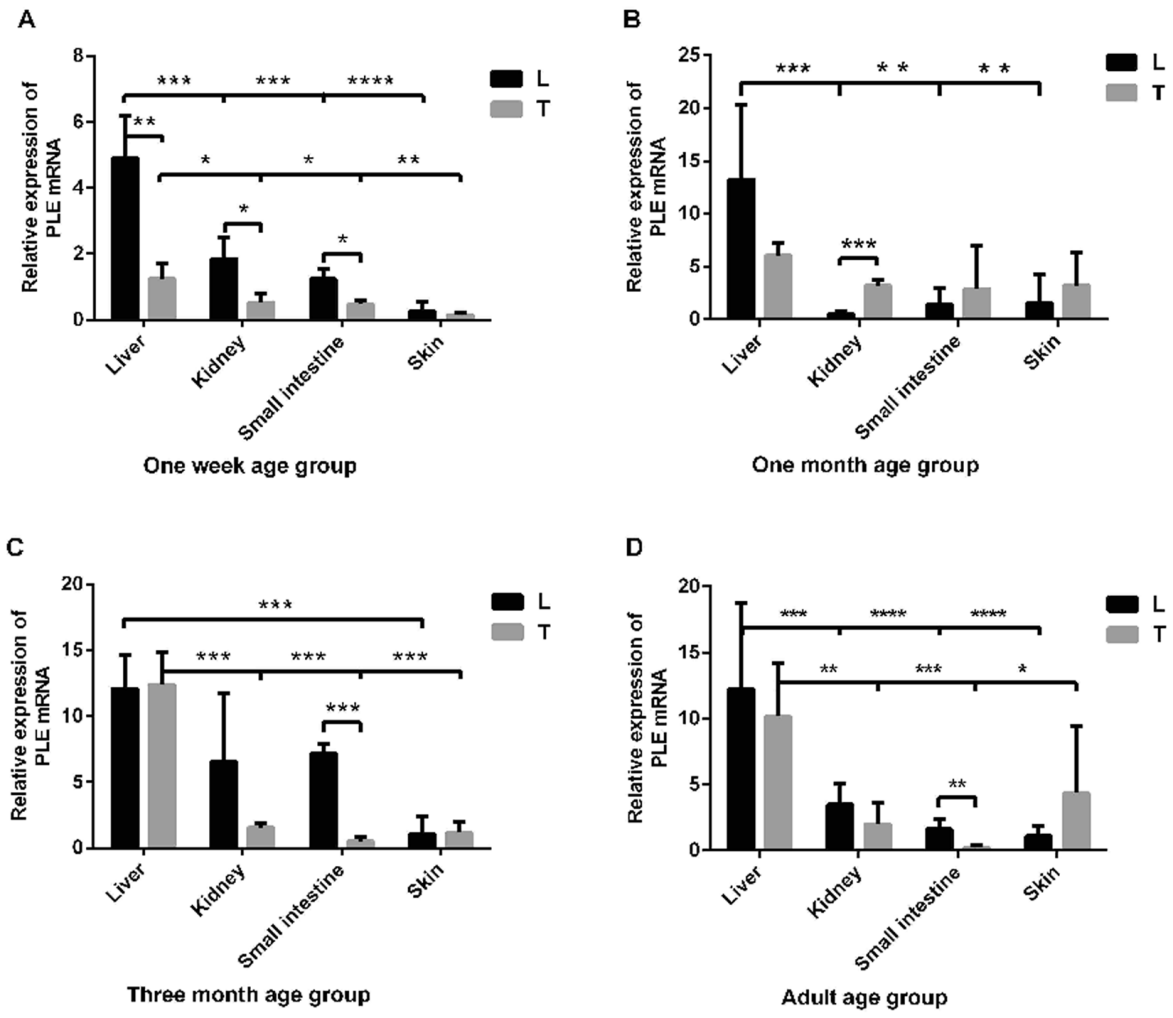
Comparison of four tissues with relatively high level of PLE in 4 age groups of LW (L) and TC pigs (T). In all the age groups, the PLE level in the liver was the highest(**A**–**D**), and compared to TC pigs, LW pigs showed a higher level of PLE mRNA in the liver (except 3-month-old pigs), kidney (except 1-month-old pigs), and small intestine (except 1-month-old pigs). (**A**) 1 week group; (**B**) 1 month group; (**C**) 3 month group; (**D**) Adult group. The data are means (±SD).The asterisk sign denotes statistical significance (*P < 0.05; **P < 0.01; *** P < 0.001; **** P < 0.0001).

In this study, PLE mRNA from various tissues (liver, kidney, small intestine, skin, fat, lung, brain, heart, spleen, muscle, lymph node and thymus) from 1-month-old and adult pigs of two breeds were tested, then PLE mRNA in liver, kidney, small intestine and skin from 1-week- and 3-month-old pigs were tested; the results are summarized in Figs 1 and 2.

The highest level of PLE mRNA in both 1-month-old and adult pigs were detected in the liver, which was followed by the kidney, small intestine, skin, fat, and lung; little PLE mRNA was detected in the brain, heart, spleen, muscle, lymph node, and thymus (Fig. 1).

The mRNA level in the liver, kidney, small intestine, and skin of the four age groups (1week, 1month, 3month, and adult pigs) was compared between breeds and among tissues. In all the age groups, the PLE level in the liver was the highest, and compared to TC pigs, LW pigs showed a higher level of PLE mRNA in the liver(except 3-month-old pigs),kidney(except 1-month-old pigs), and small intestine(except 1-month-old pigs) (Fig. 2).

### The prepared antibody against PLE has qualified specificity

**Figure 3:**
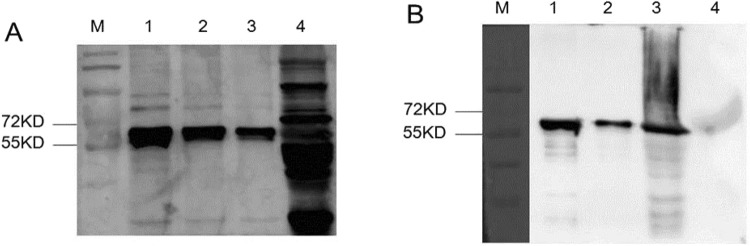
The prepared antibody against PLE has qualified specificity. The blots were incubated with non-purified rabbit antiserum against PLE (**A**) or purified rabbit antiserum against PLE (**B**). (**A**) The non-purified antiserum can bind to more than the expected component in the liver S9. M, mark; 1, 10 μg recombinant PLE; 2, 5 μg recombinant PLE; 3, 2.5 μg recombinant PLE; 4, 20 μg liver S9. (**B**) The purified antibody against PLE has qualified specificity. M, mark; 1, 5 μg recombinant PLE; 2, 1.25 μg recombinant PLE; 3, 20 μg liver S9; 4, 20 μg BSA.

The specificity of the non-purified and purified antiserum against PLE was detected with the recombinant PLE, pooled liver S9 samples and BSA. As shown in Fig. 3A, the non-purified antiserum can bind the purified recombinant PLE proteins and the positive band was about 60 kDa, while the non-purified antiserum can bind to more than the expected component of the liver S9. As shown in Fig. 3B, the purified antibody detected a single protein both in the recombinant PLE and in the pooled liver S9 samples, but detected no band in BSA, which proved that the purified antibody against PLE has qualified specificity.

### PLE level and enzyme activity in the liver of two breeds and four age groups

**Figure 4:**
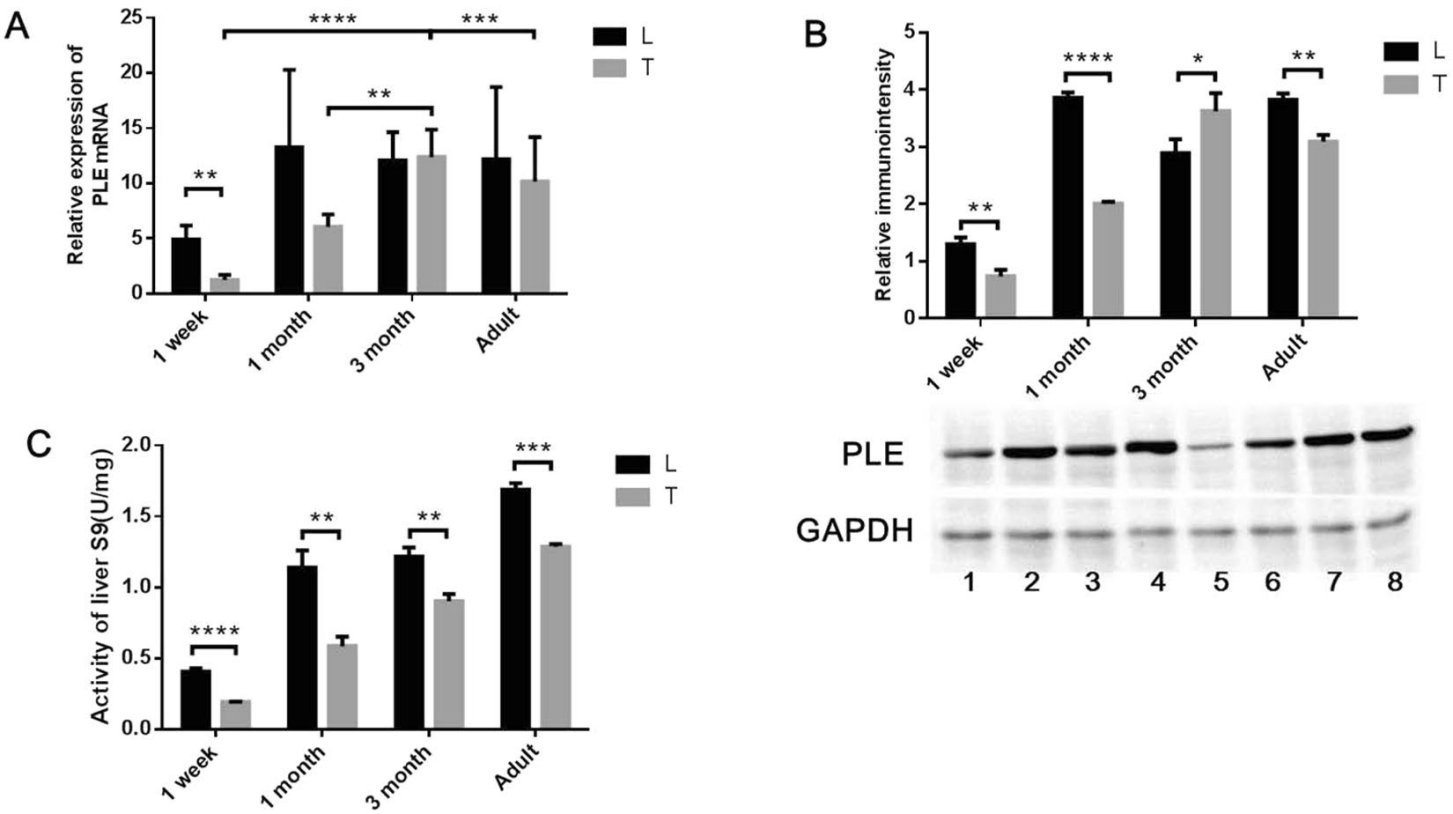
Expression level and hydrolysis activity of PLE in liver of LT (L) and TC (T). The PLE level showed a pattern positively related with age in both breeds of pigs in mRNA level (**A**), protein level (**B**) (except 1-month-old LW pigs and 3-month-old TC pigs), and enzyme activity level (**C**).The data were showed as the means ± SD. The asterisk sign denotes statistical significance (*P < 0.05; **P < 0.01; ***P < 0.001; ****P < 0.0001).

The PLE level showed a pattern positively related with age in both breeds of pigs in mRNA level (Fig. 4A), protein level (Fig. 4B) (except 1-month-old LW pigs and 3-month-old TC pigs), and enzyme activity level (Fig. 4C). In both breeds, PLE level and PLE enzyme activity level increased much more from 1-week to 1-month than among the other age groups.

In regard to mRNA level, LW pigs showed higher PLE mRNA level than that in TC pigs (except 3-month-old pigs), and PLE mRNA in 1-week-old LW pigs was 2.5 fold higher than that in TC pigs. As for protein level, PLE in LW pigs was significantly higher than the PLE in TC pigs (except for 3-month-old pigs). With respect to activity level, PLE in LW pigs was significantly higher than the PLE in TC pigs, which is consistent with the result observed for protein level (except for 3-month-old pigs).

### Cloning and analysis of PLE’s open reading frame (ORF) sequences

At least 20 PLE ORF clones from each pig liver were cloned and sequenced. All cloned sequences were translated into AA sequences according to the correct reading frame by Primer Premier5.0, referring to the methods of Brüsehaber *et al*.^28^. The PLE subtype was classified according to AA type in 25 variable sites and a total of 108 kinds of PLE isoenzymes were obtained. Except for 5 isoenzymes, which had already been reported, 103 isoenzymes were new isoenzymes. At least 50 PLE isoenzymes were present two times or more in pigs; among these, 39 PLE isoenzymes were present in more than two pigs, 19 PLE isoenzymes were expressed in both breeds. 55 PLE high-frequency isoenzymes were classified into seven categories, A to G, based on the similarity of AAs in the 25 variable sites and the members of each category were distinguished by Arabia digital; they are PLE-A1~A9, PLE-B1~B9, PLE-C1~C6, PLE-D1~D5, PLE-E1~E4, PLE-F1~F6, and PLE-G1~G16, respectively (detailed in Table 1).

**Table 1:** Different AA sequences of PLE isoenzymes (AAs differ from PLE-A1 are bold).

AA #					A									B							C						D				E					F											G								
**73**	D	D	D	D	D	D	D	D	D	**E**	**E**	**E**	**E**	**E**	**E**	**E**	***G***	**E**	**E**	**E**	**E**	**E**	**E**	**E**	D	**E**	**E**	**E**	**E**	**E**	**E**	**E**	**E**	D	D	D	D	D	D	D	D	D	D	D	D	D	D	D	D	D	D	D	D	D	D
**75**	V	V	V	V	V	V	V	V	V	**I**	**I**	**I**	**I**	**I**	**I**	**I**	**I**	**I**	**I**	**I**	**I**	**I**	**I**	**I**	V	**I**	**I**	**I**	**I**	**I**	**I**	**I**	**I**	V	V	V	V	V	V	V	V	V	V	V	V	V	V	V	V	V	V	V	V	V	V
**76**	V	V	V	V	V	V	V	V	V	**G**	**G**	**G**	**G**	**G**	**G**	**G**	**G**	**G**	**G**	**G**	**G**	**G**	**G**	**G**	V	**G**	**G**	**G**	**G**	**G**	**G**	**G**	**G**	**A**	**A**	**A**	**A**	**A**	**A**	**A**	**A**	**A**	**A**	**A**	**A**	**A**	**A**	**A**	**A**	**A**	**A**	**A**	**A**	**A**	**A**
**77**	E	E	E	E	E	E	E	E	E	**G**	**G**	**G**	**G**	**G**	**G**	**G**	**G**	**G**	**G**	**G**	**G**	**G**	**G**	**G**	E	**G**	**G**	**G**	**G**	**G**	**G**	**G**	**G**	**G**	**G**	**G**	**G**	**G**	**G**	**G**	**G**	**G**	**G**	**G**	**G**	**G**	**G**	**G**	**G**	**G**	**G**	**G**	**G**	**G**	**G**
**80**	T	T	T	T	T	T	T	T	T	**L**	**L**	**L**	**L**	**L**	**L**	**L**	**L**	**L**	**L**	**L**	**L**	**L**	**L**	**L**	T	**L**	**L**	**L**	**L**	**L**	**L**	**L**	**L**	T	T	T	T	T	T	T	T	T	T	T	T	T	T	T	T	T	T	T	T	T	T
**87**	G	G	G	G	G	G	G	G	G	G	**R**	**R**	**R**	**R**	**R**	**R**	**R**	**R**	**R**	**R**	**R**	**R**	**R**	**R**	G	**R**	**R**	**R**	**R**	**R**	**R**	**R**	**R**	**R**	**R**	**R**	G	**R**	**R**	**R**	G	G	**R**	G	G	G	G	G	G	G	G	G	G	**R**	**R**
**92**	T	T	T	T	T	T	T	T	T	T	**I**	**I**	**I**	**I**	**I**	**I**	**I**	**I**	T	T	T	T	T	T	**I**	**I**	T	T	**I**	**I**	**I**	**I**	**I**	**I**	**I**	**I**	**I**	**I**	**I**	**I**	**I**	**I**	**I**	**I**	**I**	**I**	**I**	**I**	**I**	**I**	**I**	**I**	**I**	T	**I**
**93**	L	L	L	L	L	L	L	L	L	L	**P**	**P**	**P**	**P**	**P**	**P**	**P**	**P**	**P**	**P**	**P**	**P**	**P**	**P**	**P**	**P**	**P**	**P**	**P**	**P**	**P**	**P**	**P**	**P**	**P**	**P**	**P**	**P**	**P**	**P**	**P**	**P**	**P**	**P**	**P**	**P**	**P**	**P**	**P**	**P**	**P**	**P**	**P**	**P**	**P**
**129**	L	L	L	L	L	L	L	L	L	L	**V**	**V**	**V**	**V**	**V**	**V**	**V**	**V**	**V**	**V**	**V**	**V**	**V**	**V**	**V**	**V**	**V**	**V**	**V**	**V**	**V**	**V**	**V**	**V**	**V**	**V**	**V**	**V**	**V**	L	**V**	**V**	**V**	**V**	**V**	**V**	**V**	**V**	**V**	**V**	**V**	**V**	**V**	**V**	**V**
**133**	P	P	P	P	P	P	P	P	P	P	**S**	**S**	**S**	**S**	**S**	**S**	**S**	**S**	**S**	**S**	**S**	**S**	**S**	**S**	**S**	**S**	**S**	**S**	**S**	**S**	**S**	**S**	**S**	**S**	**S**	**S**	**S**	**S**	**S**	P	**S**	**S**	**S**	**S**	**S**	**S**	**S**	**S**	**S**	**S**	**S**	**S**	**S**	**S**	**S**
**134**	M	M	M	M	M	M	M	M	M	M	**T**	**T**	**T**	**T**	**T**	**T**	**T**	**T**	**T**	**T**	**T**	**T**	**T**	**T**	**T**	**T**	**T**	**T**	**T**	**T**	**T**	**T**	**T**	**T**	**T**	**T**	**T**	**T**	**T**	M	**T**	**T**	**T**	**T**	**T**	**T**	**T**	**T**	**T**	**T**	**T**	**T**	**T**	**T**	**T**
**138**	V	V	V	V	V	V	V	V	V	V	**L**	**L**	**L**	**L**	**L**	**L**	**L**	**L**	**L**	**L**	**L**	**L**	**L**	**L**	**L**	**L**	**L**	**L**	**L**	**L**	**L**	**L**	**L**	**L**	**L**	**L**	**L**	**L**	**L**	V	**L**	**L**	**L**	**L**	**L**	**L**	**L**	**L**	**L**	**L**	**L**	**L**	**L**	**L**	**L**
**139**	V	V	V	V	V	V	V	V	V	V	**A**	**A**	**A**	**A**	**A**	**A**	**A**	**A**	**A**	**A**	**A**	**A**	**A**	**A**	**A**	**A**	**A**	**A**	**A**	**A**	**A**	**A**	**A**	**A**	**A**	**A**	**A**	**A**	**A**	V	**A**	**A**	**A**	**A**	**A**	**A**	**A**	**A**	**A**	**A**	**A**	**A**	**A**	**A**	**A**
**234**	L	L	L	L	L	L	L	**F**	**F**	L	L	**F**	**F**	**F**	**F**	L	**F**	**F**	L	L	L	L	L	**F**	L	L	L	L	L	**F**	L	L	**F**	**F**	L	L	L	**F**	**F**	L	L	L	L	L	L	L	L	L	L	L	L	L	**F**	**F**	**F**
**236**	V	V	V	V	V	**A**	**A**	**A**	**A**	V	V	**A**	**A**	**A**	**A**	**A**	**A**	**A**	V	V	V	V	V	**A**	**A**	**A**	**A**	**A**	**A**	**A**	**A**	**A**	**A**	**A**	**A**	**A**	**A**	**A**	**A**	V	V	A	A	A	A	V	**A**	**A**	V	V	**A**	**A**	**A**	**A**	**A**
**247**	A	A	A	A	A	**G**	**G**	**G**	**G**	A	A	**G**	**G**	**G**	**G**	**G**	**G**	**G**	**G**	**G**	**G**	**G**	**G**	**G**	**G**	**G**	**G**	**G**	**G**	**G**	**G**	**G**	**G**	**G**	**G**	**G**	**G**	**G**	**G**	A	A	G	G	G	G	**G**	**G**	**G**	**G**	**G**	**G**	**G**	**G**	**G**	**G**
**285**	F	F	F	F	F	F	F	F	F	F	F	F	F	F	F	F	F	F	F	F	F	F	F	F	F	F	F	F	F	**P**	**P**	**P**	**P**	**P**	**P**	**P**	**P**	**P**	**P**	F	F	F	F	F	F	F	F	F	F	F	F	F	F	F	F
**286**	L	L	L	**F**	**F**	L	L	**F**	**F**	L	L	L	**F**	**F**	**F**	**F**	**F**	**F**	**F**	**F**	**F**	**F**	**F**	**F**	L	L	L	L	L	L	L	L	L	L	L	L	L	L	L	L	L	L	L	L	L	L	L	L	**F**	**F**	**F**	**F**	**F**	**F**	**F**
**287**	T	T	T	**A**	**A**	T	T	**A**	**A**	T	T	T	**A**	**A**	**A**	**A**	**A**	**A**	**A**	**A**	**A**	**A**	**A**	**A**	T	T	T	T	T	T	T	T	T	T	T	T	T	T	T	T	T	T	T	T	T	T	T	T	**A**	**A**	**A**	**A**	**A**	**A**	**A**
**290**	F	F	**L**	**L**	**L**	**L**	**L**	**L**	**L**	F	F	F	**L**	**L**	**L**	**L**	**L**	**L**	**L**	**L**	**L**	**L**	**L**	**L**	**L**	**L**	**L**	**L**	**L**	**L**	**L**	**L**	F	**L**	**L**	**L**	**L**	**L**	**L**	F	F	**L**	**L**	**L**	**L**	**L**	**L**	**L**	**L**	**L**	**L**	**L**	**L**	**L**	**L**
**294**	Q	Q	Q	**P**	**P**	Q	Q	**P**	**P**	Q	Q	Q	**P**	**P**	**P**	**P**	**P**	**P**	**P**	**P**	**P**	**P**	**P**	**P**	Q	Q	Q	Q	Q	**P**	**P**	**P**	Q	**P**	**P**	**P**	**P**	**P**	**P**	Q	Q	Q	Q	Q	Q	Q	Q	Q	**P**	**P**	**P**	**P**	**P**	**P**	**P**
**302**	P	P	**T**	**T**	**T**	P	**T**	**T**	**T**	P	P	P	**T**	**T**	**T**	**T**	**T**	**T**	P	P	**T**	**T**	**T**	P	P	P	P	**T**	**T**	**T**	**T**	**T**	P	**T**	**T**	**T**	**T**	**T**	**T**	P	P	P	P	**T**	P	P	**T**	**T**	P	**T**	**T**	**T**	**T**	**T**	**T**
**459**	F	**A**	F	F	**A**	F	F	**A**	**A**	F	F	F	F	**A**	**A**	**A**	**A**	**A**	**A**	F	F	**A**	**A**	**A**	F	F	F	F	F	F	F	**A**	**A**	F	F	**A**	**A**	**A**	**A**	F	F	F	F	F	**A**	**A**	**A**	**A**	**A**	**A**	**A**	**A**	**A**	**A**	**A**
**461**	L	**F**	L	L	**F**	L	L	**F**	**F**	L	L	L	L	L	**F**	**F**	**F**	**F**	**F**	L	L	L	**F**	**F**	L	L	L	L	L	L	L	**F**	**F**	L	L	**F**	**F**	**F**	**F**	L	L	L	L	L	**F**	**F**	**F**	**F**	**F**	**F**	**F**	**F**	**F**	**F**	**F**
**463**	K	**R**	K	K	**R**	K	K	**R**	K	K	K	K	K	K	K	**R**	**R**	**R**	K	K	K	K	K	K	K	K	K	K	K	K	K	**R**	**R**	K	K	**R**	**R**	K	**R**	K	K	K	K	K	K	K	K	**R**	K	K	K	**R**	**R**	**R**	**R**
**PLE**	A1	A2	A3	A4	A5	A6	A7	A8	A9	B1	B2	B3	B4	B5	B6	B7	B8	B9	C1	C2	C3	C4	C5	C6	D1	D2	D3	D4	D5	E1	E2	E3	E4	F1	F2	F3	F4	F5	F6	G1	G2	G3	G4	G5	G6	G7	G8	G9	G10	G11	G12	G13	G14	G15	G16

### PLE isoenzyme abundance in different age groups and breeds

**Figure 5:**
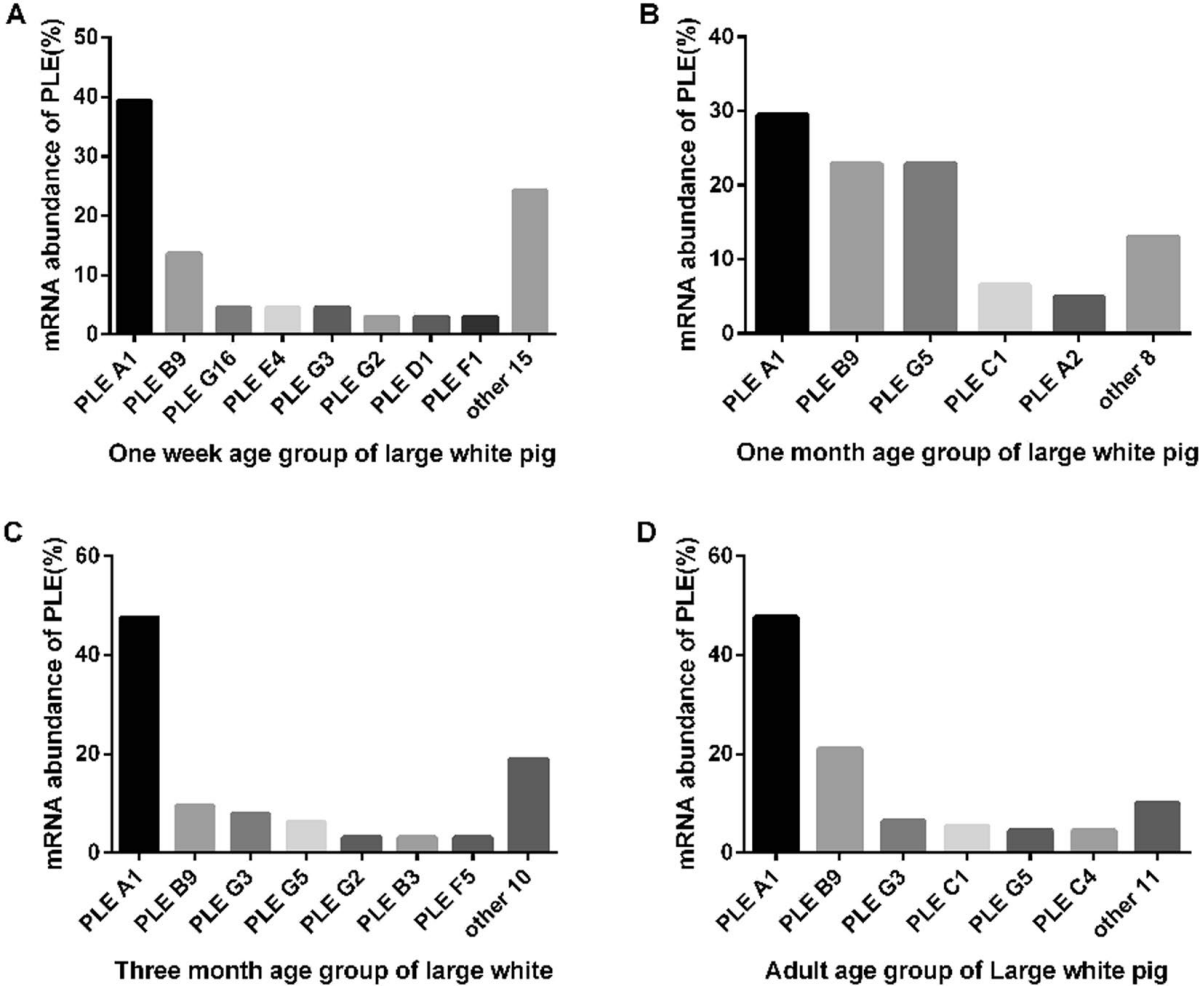
PLE isoenzyme mRNA abundance in liver in four age groups of LW pigs. (**A**–**D**) PLE-A1 was the highest and PLE-B9 was the second in four age stages. (**A**) 1-week group; (**B**) 1-month group, (**C**) 3-month group; (**D**) adult group. The PLE isoenzymes were classfied into PLEA1-G16 according to the 25 variable sites. The amount of all PLE isoenzymes and specific PLE isoenzyme in each group were counted. The percentage of specific PLE isoenzyme was calculated taking all PLE isoenzymes as 100 percent in each group, the percentage of specific PLE isoenzyme represents its mRNA abundance.

**Figure 6:**
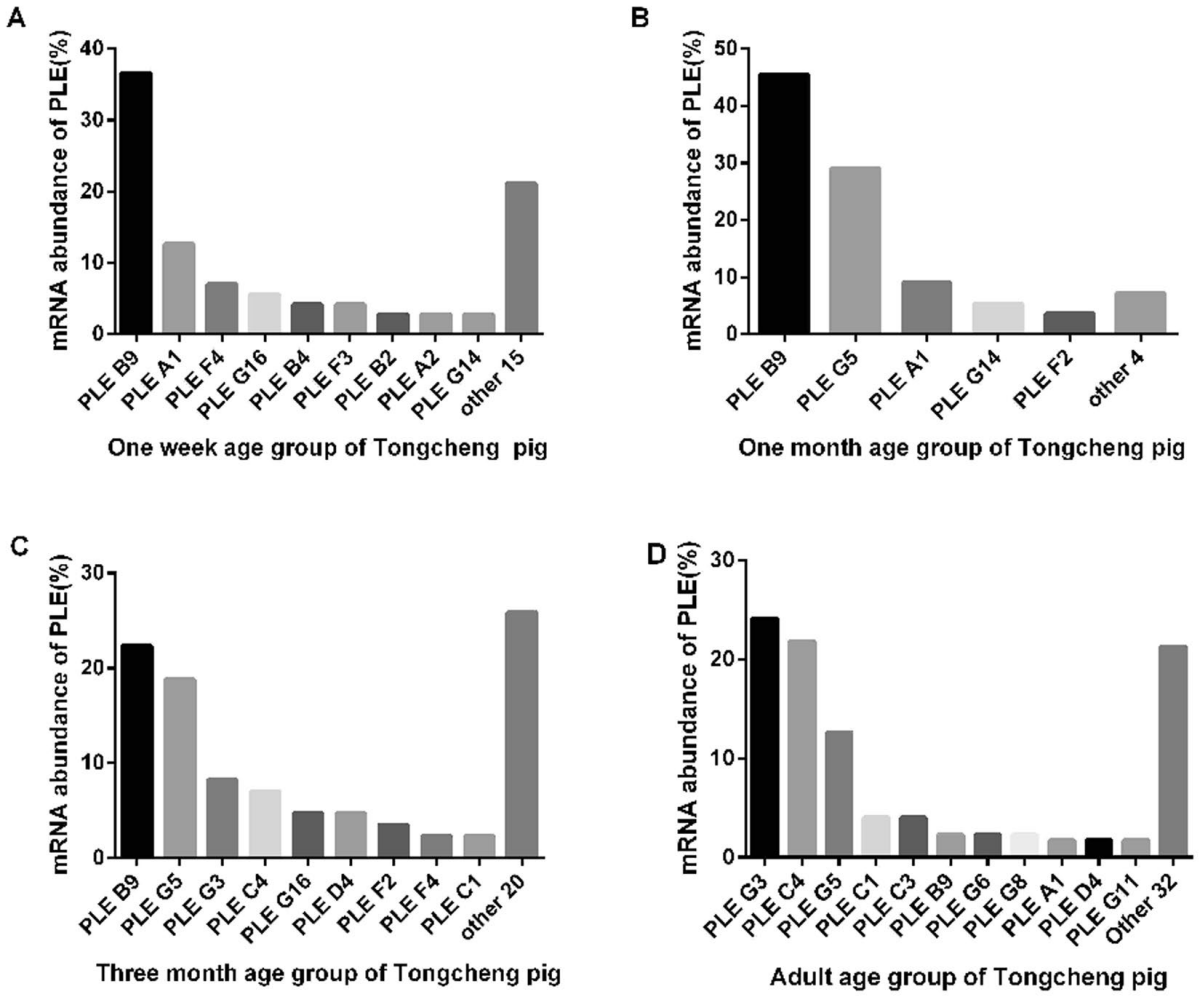
PLE isoenzyme mRNA abundance in liver in four age groups of TC pigs. (**A**–**C**)In the first three age groups, PLE-B9 was the most abundant, the second most abundant isoenzymes were PLE-A1 (**A**), PLE-G5 (**B**), PLE-G5 (**C**) in 1-week, 1-month and 3-month, respectively. (**D**) In adult pigs, PLE-G3 became the most abundant isoenzyme and PLE-C4 was the second most abundant isoenzyme. (**A**) 1 week group; (**B**) 1 month group; (**C**) 3 month group; (**D**) adult group. The PLE isoenzymes were classfied into PLEA1-G16 isozyme according to the 25 variable sites. The amount of all PLE isoenzymes and specific PLE isoenzyme in each group were counted. The percentage of specific PLE isoenzyme was calculated taking all PLE isoenzymes as 100 percent in each group, so the percentage of specific PLE isoenzyme represents its mRNA abundance.

As shown in Figs 5 and 6, not only the two different breeds presented different PLE abundance, but also different age groups presented different PLE abundance.

In LW pigs (Fig. 5), PLE-A1 was the highest and PLE-B9 was the second in four age stages, the abundance of PLE-A1/PLE-B9 were 39%/14%, 30%/23%, 48%/10%, and 48%/21% in 1week, 1month, 3month and adult pigs, respectively. In addition to PLE-A1 and PLE-B9, PLE-G categories were relatively highly abundant isoenzymes in all age groups, especially in 1-month-old pigs (23% PLE-G5).

In TC pigs, PLE isoenzyme diversity and abundance are more complex. In the first three age groups, PLE-B9(instead of PLE-A1 compared with LW pigs) was the most abundant, with 37%, 45%, and 22% in pigs aged 1-week, 1-month, and 3-month, respectively (Fig. 6A–C), while in adult pigs, PLE-G3 replaced PLE-B9 and became the most abundant isoenzyme (24%)(Fig. 6D). The second most abundant isoenzymes were PLE-A1(13%), PLE-G5(29%), PLE-G5(19%), and PLE-C4(22%) in 1-week, 1-month, 3-month and adult pigs, respectively. It should be mentioned that both PLE G and C categories increased with age.

With respect to the comparison between the two breeds, both expressed similar PLE isoenzymes (such as PLE-A1, B9, G5) and PLE-G5 surged in 1-month-old pigs. On the other hand, the two breeds showed obvious differences in PLE abundance and PLE expression profile. It is clear that PLE profile in TC pig is much more complex than that in LW pig. The number of PLE isoenzymes in TC pigs is much higher than that in LW pigs, particularly in older groups. For example, in 3-month-old TC and LW pigs, the different PLE isoenzymes were 29 and 17, respectively, while in the adult groups, they were 43 and 17, respectively. In LW pigs, PLE-A1 was the most abundant isoenzyme in all age groups, while in TC pigs, PLE-B9 was the most abundant isoenzyme in pigs aged 1-week, 1-month and 3-month. PLE-G3 was the most abundant isoenzyme in adults. Compared to 1-month-old pigs, although PLE-B9 decreased by about 50% in 3-month-old pigs of both breeds, in the adult group, PLE-B9 recovered in LW pigs, while in TC pigs PLE-B9 continually decreased from 22% to 2%, and both PLE-G3 and PLE-C4 increased by about 15% and was substituted for PLE-B9. Although PLE-G5 surged in 1-month-old pigs of both breeds and then decreased with age, its expression level was higher and it decreased to a lesser extent in TC pigs.

## Discussion

In order to ensure the data obtained in this study is representative and practical, LW pig and TC pig-China’s national protected breed- were selected as experimental animals in this study, and PLE expression profile and breed differences were explored in mRNA, protein and enzyme activity levels. In order to recover the actual expression profile, referring to human age, four age groups—1-week, 1-month, 3-month and adult pigs—were selected. For the representativeness of each of the two breeds, 3 individuals without blood relationship through at least three generations were selected for each age group.

Because of the high similarity of cDNA sequence of PLE isoenzymes, it is very difficult to design a probe to distinguish different PLE isoenzymes. Thus, PLE universal primers were designed to detecte the total PLE level according to the reserved sequence of PLE cDNA, a large amount of PLE ORF in the liver were cloned and sequenced, and the abundance of specific isoenzyme at the mRNA level was obtained.

Similarly, because of the high degree of AA sequence homology, it is also very difficult to develop an antibody against PLE-specific isoenzyme. This is why only the PLE universal antibody was prepared in this study and WB was used to detect porcine liver overall PLE protein level; thus, the protein level of each particular PLE isoenzyme can only be estimated from mRNA abundance.

We found the highest level of mRNA in the liver, followed by kidney, intestine and skin. The liver and kidney play a major role in the body’s metabolism, detoxification, and excretion, suggesting that PLE may, through hydrolysis, play an important role in drug metabolism and detoxification in the liver, as well as in substance removal or reabsorption in the kidney. The small intestine is the main organ for absorption of nutrients and other exogenous substances; intestinal PLE may be related to the absorption of esters in the intestine. As reported, mice carboxylesterases1 in intestine can regulate assembly, secretion, and clearance of chylomicrons^10^. Some gut harmful toxic substances may also be hydrolyzed and detoxified by PLE. The skin acts as a natural barrier of the body; the skin PLE may be related to detoxification of some toxic substances. In conclusion, tissue and organ distribution characteristics of PLE suggested that PLE not only plays an important role in the metabolism of ester drugs but also plays an important role in some physiological processes and in the process of self-protection.

In this study, more than 800 PLE cDNA positive clones from the liver of 24 individuals of two different breeds in four age groups were obtained and sequenced. According to the classification method of Brüsehaber^28^, 108 isoenzymes of PLE were registered, among which were the previously reported PLE1, PLE2, PLE5, PLE 6 and APLE. We found 39 isoenzymes in more than two individuals and 19 were found in the two breeds. In one individual, there were as many as about 15 isoenzymes; considering that active PLE is a random trimer of any subunit, there will be as many as about 120 PLE isoenzymes in one individual, which suggested that the roles of PLE in pigs should not be ignored. Since the number of new found PLE isoenzymes is far more than the 7 previously reported ones, thus, according to the similarity of 25 variable sites on AAs, the 55 high frequency isoforms were divided into 7 groups (A~G), and within the same group, different members were specified by Arabia digital. A blast search of cDNA sequence with the 13th to 14th exons of PLE in NCBI yielded 14 PLE sequences of cDNA, and 14 PLE isoenzymes (Supplementary material) were also found in this study, which suggests the credibility of the PLE isoforms found in this study.

The two breeds of pigs under study showed obvious differences both in PLE abundance and PLE profile. In order to explore the significance of these differences, major PLE isoenzymes (PLE-A1, B9, G3, G5, C4, and others) were expressed, and the activities of these recombinant PLEs were measured by p-nitrophenyl acetate (p-NPA), which is the universal substrate for PLE. The results showed that the hydrolysis activity of PLE-B9 was the highest, which was about two-fold that of PLE-A1 and about four-fold that of PLE-G3, PLE-G5, and PLE-C4 (submitted). Combining the differences in PLE total amount, isoenzyme type, hydrolysis activity, and abundance between breeds and among age groups, if one specific clinical pig drug could be hydrolyzed by PLE, it is reasonable to speculate that the hydrolysis of the drug would be different between TC and LW pigs and these differences will lead to differences in efficacy and toxicity of the same drug in two breeds and at different ages. If hydrolysis of the drugs abolished or decreased the drug efficacy, TC pigs would be more vulnerable than LW pigs, and vice versa. Furthermore, the differences may also explain why PLE hydrolysis activity was not always consistent with mRNA levels and protein abundance.

For both breeds, PLE-G5 increased significantly with growth (1-month to 3-month), and PLE-G3 increased significantly during maturity (adult), suggesting that PLE-G3 and PLE-G5 may play an important role during growth and maturity, respectively. The significance and regulation mechanism of the profile observed need further study.

The higher level and activity of PLE in the livers of LW pigs than in TC pigs may lead to the higher metabolism level of related compounds, which probably explain the higher growth rate of the LW pigs. Carboxylesterases1 gene deletion reportedly leads to obesity and fatty liver in mice^10^, which suggested that a lower PLE level may be the cause of the higher fat accumulation rate in TC pig. it was also reported that the same pathogen PRRSV induced obvious differences of inflammation response between TC and LW pigs, and the LW pig showed a much higher inflammatory response against PRRSV than TC^45^, which suggested that PLE probably play an important role in inflammation response procedure through hydrolysis of some inflammatory factors.

However, because of the limits of pigs population, the same amount of male and female pigs in each group which didn’t have blood relationship through at least three generations were difficult to satisfy. So the gender differences of PLE weren’t researched. In addition, according to the report of Zhu *et al*.^24^ that sex seems to be an unlikely factor contributing to the regulation of carboxylesterase 1 and carboxylesterase 2 in human and mouse, it suggested that the gender differences of pig may not impact on the level of PLE very much. Because of the high degree of sequence homology and analogously physical and chemical properties among PLE isoenzymes, the antibody against specific PLE isoenzyme was difficult to be developed and a purified PLE isoenzyme to be obtained by physical or chemical methods. So the protein level and enzymatic activity of specific PLE isoenzyme was not explored. As demonstrated in this study, PLE showed obvious age and breed difference between TC and LW pigs, but the mechanisms underlying need further studied. In addition, whether PLE could catalyze the hydrolysis of some important pig drugs and affect the drugs clinical efficacy accordingly, the breed difference lead to clinical efficacy difference also need further study.

In conclusion, the expression level of PLE is closely related to pig age, and there are obvious differences between the LW and TC breeds, which suggests that clinical medication should be adjusted not only according to the age of the pig but also according to the breed. Furthermore, the growth rate and fat accumulation rate in pigs may be related to PLE, and thus, PLE can be used as a reference target in pig breeding.

## Methods

### Animals

In order to ameliorate the suffering of the pigs and the rabbits, this experiment was performed in strict accordance with the Guide for the Care and Use of Laboratory Animals Monitoring Committee of Hubei Province, China, and the protocol was approved by the Committee on the Ethics of Animal Experiments at the College of Veterinary Medicine, Huazhong Agricultural University.

New Zealand white rabbits were obtained from the Laboratory Animal Center of Huazhong Agricultural University. LW pigs were purchased from Hebei Tianzhong Animal Husbandry Co., Ltd., Hubei Province, China. TC pigs were purchased from conservation farms in Tongcheng County, Hubei Province, China. All pigs were fed under the same conditions. The information of the pigs used in this study was summarized in Table 2. The livers, small intestine, lung, kidney, etc., were sampled from 1-week, 1-month, 3-month, and adult pigs, respectively. These tissues were immediately frozen in liquid nitrogen for preparing total RNA and the liver tissues were also used later for preparing S9 fractions.

**Table 2:** The information of the pigs used in this study.

Breeds/Age group		1-week	1-month	3-month	adult
LW	sex	male	male	male	female
	number	3	3	3	3
TC	sex	male	male	female	female
	number	3	3	3	3

The individuals of each age group didn’t have blood relationship through at least three generations.

### RNA isolation and qRT-PCR

Tissues from fresh pig liver and other organs (1 g) were homogenized and poly(A) RNA was isolated using Trizol reagent (Invitrogen, Carlsbad, CA, USA) according to the manufacturer’s instructions. The SYBR Premix Ex Taq Kit (TaKaRa,DaLian, China) was used to conduct qRT-PCR. Relative mRNA levels were calculated by applying the 2^−ΔCt^ method using reference gene GAPDH. The sequences for the primers were as follows:

PLE sense, 5′- GGGGATGTGGTGTTTGGT-3′

PLE anti-sense, 5′-TGGGTTTCTTGTCCGATG -3′

GAPDH sense, 5′-GAAGGTCGGAGTGAACGGAT-3′

GAPDH anti-sense, 5′-CATGGGTAGAATCATACTGGAACA-3′

The PCR amplification was conducted in a total volume of 20 µL. Amplification and quantification were done with the BIO-RAD iQ5 Real-Time PCR System (BIO-RAD, Hercules, CA).

### Antibody preparation

After analyzing the AA sequences of 5 PLE isoenzymes (PLE1-PLE5) and the rabbit carboxylesterase AA sequences, two peptide sequences that were preserved in pigs but different in rabbits were synthesized by Shanghai Top-Peptide Bio Co. Ltd. (Shanghai, China). The sequences were PLE-C1(SKEAAKKPPKIKC) and PLE-C2 (CNTQAAKRLKGEE). They were mixed in equal amounts and then conjugated to keyhole limpet hemocyanin. Anti-peptide antibodies were raised in New Zealand white rabbits. The immunization and antibody purification procedures were conducted as described previously^46^. The antibody was purified by Willget biotech Co., Ltd. (Shanghai, China).

### Preparation of liver S9 and enzymatic assays

The liver S9 fractions were prepared by centrifugation of liver homogenates at 9000 × *g* for 20 min at 4 °C and the liver S9 samples were prepared by equally pool of the same age group. The hydrolytic activities of pool S9 samples for p-NPA (Aladdin, Shanghai, China) were assayed as described previously^47^. Sample cuvettes (1 mL) contained 25 to 250 μg of liver S9 samples at pH 7.2, 50 mM PBS, and 1 mM p-NPA, at room temperature. Reactions were initiated by adding p-NPA (10 μL of 100 mM stock in acetonitrile); the hydrolytic rate was recorded from an increase in absorbance at 400 nm by a microplate reader (BIO-TEK, ELX800, VT, USA). The extinction coefficient (E400) was determined to be 13 mM^−1^ cm^−1^. Then the production of para-nitrophenyl were calculated by the Lambert beer’s law formula: ΔA = Ƹ *ΔC*L(ΔA, change in absorbance. Ƹ, the extinction coefficient. ΔC, change in sample concentration. L, optical path.). Enzymatic activity was calculated according to the definition of a unit of enzyme activity: the amount of enzyme required to convert 1 mol p-NPA into para-nitrophenyl per min.

### Western blot analysis

The liver S9 pool samples were used for Western blot, and recombinant PLE expressed by our research group were used as control. The primary antibody (Ab) was the Ab against PLE prepared by this study. The Ab against GAPDH was purchased from Proteintech (Wuhan, China). The second Ab was goat anti-rabbit IgG conjugated with horseradish peroxidase from Wuhan booster biotechnology company (Wuhan, China). Western blot analysis was conducted as previously described^46^. Samples were resolved by 7.5% SDS-PAGE in a mini-gel apparatus and transferred electrophoretically to a PVDF membrane. The blots were incubated with the primary Abs respectively. The signal was captured by MF-Chemi BIS Chemiluminescence imager(Bio-Imaging Systems). And the relative intensities were quantified by Image J software.

### PLE ORF cloning, sequencing, and analyzing

The PLE ORF was cloned by the T-A cloning technique. Total RNA was extracted. The cDNA synthesis by RT-PCR was performed using M-MLV (TAKARA, DaLian, China) with an oligo (dT) primer(TAKARA, DaLian, China) following the protocol provided with the kit. RT-PCR products were used as templates for the amplification of the complete PLE ORF by two gene-specific primers according to the sequence of PLE cDNA (X63323):

PLE-F:5′-ATGTGGCTTCTCCCGCTGGTCCTGA-3′

PLE-R:5′-TCACTTTATCTTGGGTGGCTTCTTT-3′

The PCR products were detected and separated by 1% agarose gel electrophoresis and then connected to the pMD18-T vector (TaKaRa, DaLian, China). The ligation products were transformed into *E*.* coli DH5a* (TaKaRa, DaLian, China) and at least 20 positive clones from each pig were sequenced by Tsingke (Wuhan, China). A total of over 800 clones were sequenced from the livers of 24 pigs.

All the sequences of PLE ORF were translated into AA sequences by Primer Premier 5.0. The translated AA sequences were aligned by the ClustalX2 software and then classified into PLE A to PLE G according to the 25 variable AA sites, then the mRNA abundance of each PLE isoenzyme in the liver of the four age groups and two breeds was calculated.

### Statistical Analysis

The data were expressed as mean ± standard deviation (SD). And the mean, SD and P values of PLE mRNA level, protein level and enzyme activity were calculated by GraphPad Prism 6.01.

## Electronic supplementary material

Supplementary Information
