# Supplementary material for: Breed Differences in Pig Liver Esterase (PLE) between Tongcheng (Chinese Local Breed) and Large White Pigs
Source: Sci Rep. 2018 Nov 5;8:16364. doi: 10.1038/s41598-018-34695-y (PMC6218520; doi:10.1038/s41598-018-34695-y)
Supplement: Supplementary file 1 — Supplementary Information [file 41598_2018_34695_MOESM1_ESM.pdf]

title page

Breed Differences in Pig Liver Esterase (PLE) between Tongcheng (Chinese local breed) and Large White Pigs

Qiling Xiao[1,2,3], Qiongqiong Zhou[1,2,3], Lu Yang[1,2,3], Zhongyuan Tian[1,2,3], Xiliang Wang[1,2,3], Yuncai Xiao[1,2,3], Deshi Shi[1,2,3,*]

[1]State Key Laboratory of Agricultural Microbiology, College of Veterinary Medicine, Huazhong Agricultural University, Wuhan, 430070, Hubei, China

[2]Key Laboratory of Development of Veterinary Diagnostic Products of Ministry of Agricultural, College of Veterinary Medicine, Huazhong Agricultural University, Wuhan, 430070, Hubei, China

[3] The Cooperative Innovation Center for Sustainable Pig Production, Wuhan, 430070, Hubei, China

[*]Corresponding author: Deshi Shi, rock@mail.hzau.edu.cn

Supplementary Figure S3

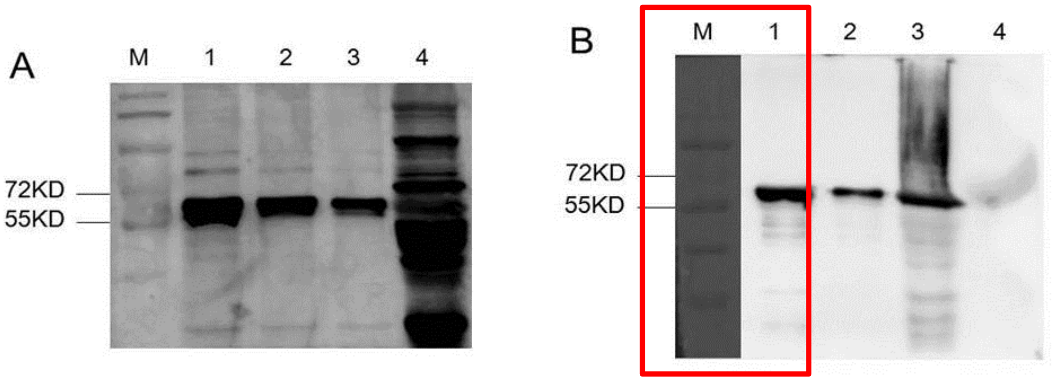

Figure 3. The prepared antibody against PLE has qualified specificity.

The following Figure is the original image of Figure 3A.

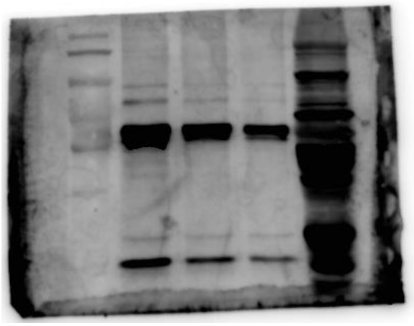

Figure 3AS

In the Figure 3B, The marker was captured through the Preview Mode because it couldn't developed without chemiluminescent substrate. So this figure was cropped from the following two figures which came from the same gel.

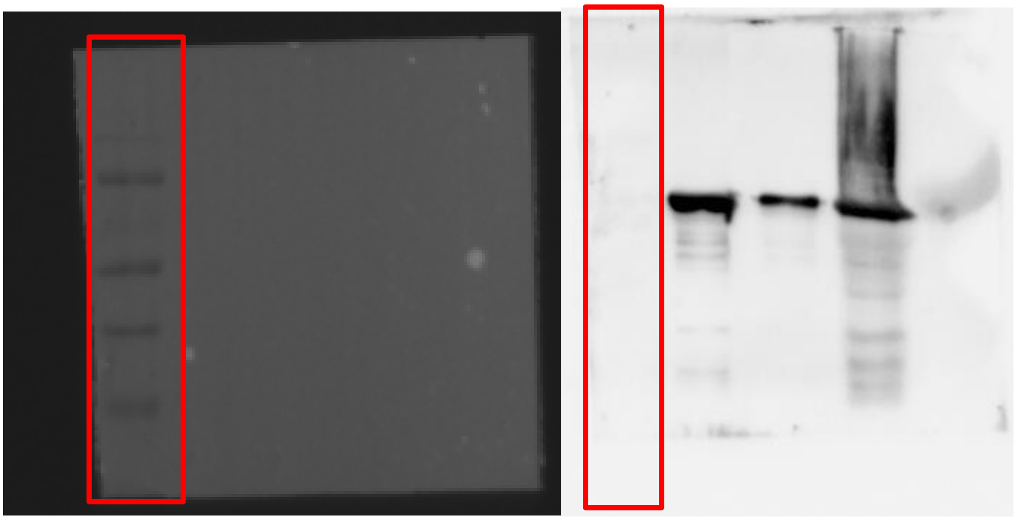

Figure 3BS

Supplementary Figure S4

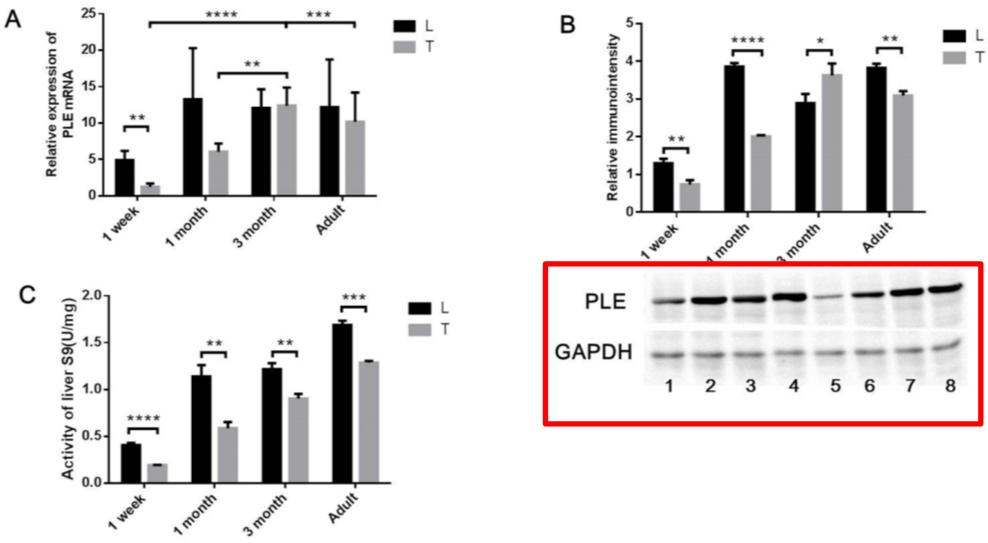

Figure 4. Expression level and hydrolysis activity of PLE in liver of LT (L) and TC(T).

In the Fig. 4B, the grouping of blots cropped from different parts which were in the red boxes of the following figure. The line 1 and line 2 were loaded the same S9 sample that was prepared by 1 week Large White pigs, but the loading quantity of sample were different.

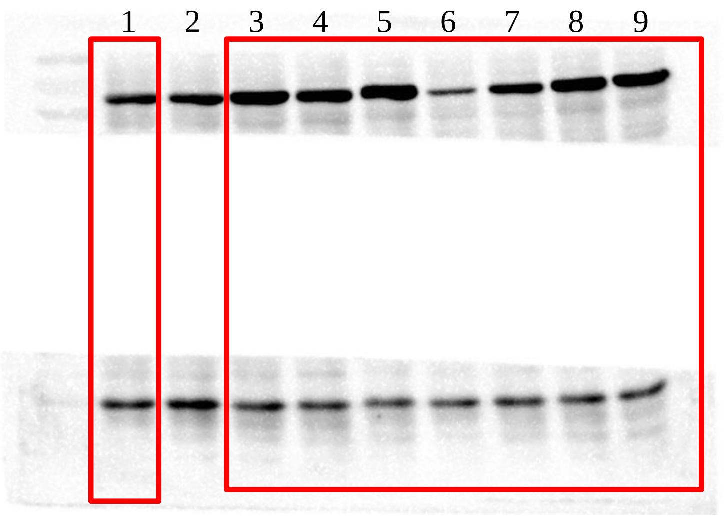

Supplementary Discussion

Amino acid #	11555<br>1247	28878<br>5111	37192<br>2719	11555<br>4036	37192<br>0273	11555<br>2331	16441<br>4677	37191<br>4319	28878<br>4728	4752<br>3571	3831<br>578	37192<br>2722	54582<br>9765	1930
**73**	E	E	E	E	E	G	E	D	D	D	D	D	D	D
**75**	I	I	I	I	I	I	I	V	V	V	V	V	V	V
**76**	G	G	G	G	G	G	G	A	A	A	A	A	V	V
**77**	G	G	G	G	G	G	G	G	G	G	G	G	E	E
**80**	L	L	L	L	L	L	L	T	T	T	T	T	T	T
**87**	R	R	R	R	R	R	R	R	R	G	G	G	G	G
**92**	T	T	T	T	T	T	I	I	I	I	I	I	T	T
**93**	P	P	P	P	P	P	P	P	P	P	P	P	L	L
**129**	V	V	V	V	V	V	V	V	V	V	V	V	L	L
**133**	S	S	S	S	S	S	S	S	S	S	S	S	P	P
**134**	T	T	T	T	T	T	T	T	T	T	T	T	M	M
**138**	L	L	L	L	L	L	L	L	L	L	L	L	V	V
**139**	A	A	A	A	A	A	A	A	A	A	A	A	V	V
**234**	L	L	L	L	L	L	F	F	F	L	L	L	L	L
**236**	V	V	V	V	V	V	A	A	A	A	A	A	V	V
**247**	G	G	G	G	G	G	G	G	G	G	G	G	A	A
**285**	F	F	F	F	F	F	F	P	P	F	F	F	F	F
**286**	F	F	F	F	F	F	F	L	L	L	L	L	L	L
**287**	A	A	A	A	A	A	A	T	T	T	T	T	T	T
**290**	L	L	L	L	L	L	L	L	L	L	L	L	F	F
**294**	P	P	P	P	P	P	P	P	P	P	P	Q	Q	Q
**302**	T	T	T	T	P	P	T	T	P	P	P	T	P	P
**459**	A	A	A	A	A	A	F	F	F	F	F	F	F	F
**461**	F	F	F	F	F	F	L	L	L	L	L	L	L	L
**463**	K	K	K	K	K	K	K	K	K	K	K	K	K	K
	C5	C5	C5	C5	C1	C1	B4	F1	F1	G	G	G5	A1	A1

A blast search of cDNA sequence with the 13th to 14th exons of PLE in NCBI yielded 14 PLE sequences of cDNA, it is amino acid pattern of the 14 PLE Isoenzymes above and the 14 PLE isoenzymes were also found in this study, which suggests the credibility of the PLE isoforms found in this study.
